# QuantiFERON-TB gold test: a valuable diagnostic tool for tubercular uveitis in non-endemic regions

**DOI:** 10.1007/s15010-026-02789-9

**Published:** 2026-04-17

**Authors:** Sebastian Albus, Antonia Koch, Uwe Pleyer, Anne Rübsam, Leif E. Sander, Martin Witzenrath, Nikolai Menner, Lynn S. zur Bonsen, Dominika Pohlmann

**Affiliations:** 1 Fächerverbund Für Infektiologie, Pneumologie Und Intensivmedizin, Charité –Universitätsmedizin Berlin, Corporate member of Freie Universität Berlin and Humboldt- Universität Zu Berlin, Augustenburger Platz 1, 13353 Berlin, Germany; 2 Department of Ophthalmology, Charité– Universitätsmedizin Berlin, corporate member of Freie Universität Berlin and Humboldt- Universität Zu Berlin, Augustenburger Platz 1, 13353 Berlin, Germany; 3 Berlin Institute of Health at Charité – Universitätsmedizin Berlin, Charitéplatz 1, 10117 Berlin, Germany

**Keywords:** COTS, Quantiferon, Tuberculosis, Tubercular uveitis, Uveitis

## Abstract

**Purpose:**

To investigate the diagnostic challenges of tubercular uveitis, given the frequent absence of pulmonary involvement and the limited accuracy of ocular detection methods.

**Methods:**

We conducted a retrospective analysis of real-world data from 2285 uveitis patients who underwent QFT screening between 2013 and 2022 at a tertiary care centre in Germany.

**Results:**

Among the 2285 screened patients, 172 (7.5%) uveitis patients tested QFT-positive. 17 patients were diagnosed with clinically active tuberculosis disease (TBD) and 155 patients with clinically inapparent tuberculosis infection (TBI). TBD patients were younger (40 vs. 58 years; *p* = 0.005), more likely to originate from Southeast Asia region (56.3% vs. 43.7%; *p* < 0.005), and more frequently presented with clinical symptoms (17.6% vs. 1.3%; *p* < 0.005). No differences were observed in the prevalence of tuberculosis (TB)-suspicious findings on chest X-ray (35.3% vs. 17%; *p* = 0.08) or CT (71.4% vs. 31.6%; *p* = 0.06). None of the 98 sputum samples yielded a positive result. Microbiological confirmation was achieved in 4 cases via bronchoscopy. Among TBI patients (*n* = 155), idiopathic inflammatory uveitis was diagnosed in 109 cases (70.3%), while an alternative aetiology was identified in 46 (29.7%). Posterior uveitis was the predominant subtype in both groups (TBD 65.6% vs. TBI 40.8%; *p* = 0.03). In TBD patients, the prevalence of bilateral involvement was significantly higher (93.8% vs. 64.0%; *p* = 0.001), and active retinal vasculitis was significantly more common (75.5% vs. 14.5%; *p* < 0.005).

**Conclusion:**

This study highlights the importance of TB screening, even in non-endemic regions, as evidenced by the prevalence of QFT positivity and active TB cases in uveitis patients.

**Supplementary Information:**

The online version contains supplementary material available at 10.1007/s15010-026-02789-9.

## Introduction

Tuberculosis (TB) remains one of the major infectious diseases worldwide [1]. While pulmonary TB is the best known presentation, extrapulmonary TB is common and causes significant morbidity and mortality [2–5]. Ocular TB occurs in approximately 1.5% of patients with confirmed systemic TB [6, 7]. Globally, the prevalence of tubercular uveitis among all uveitis cases is estimated at 4%, with rates up to 22–48% in TB-endemic countries [8, 9]. However, the true prevalence is likely underestimated due to diagnostic challenges, including the paucibacillary nature of extrapulmonary TB and limitations of current microbiological and molecular tests in the detection of *Mycobacterium tuberculosis* (MTB) both in the eye and elsewhere [10, 11]. Tubercular uveitis can present with a range of phenotypes, most commonly multifocal choroiditis, solitary choroidal nodules, anterior granulomatous uveitis, and retinal vasculitis [6, 12].

Uveitis has been reported both in patients with tuberculosis disease (TBD) and in individuals with immunologic evidence of tuberculosis infection (TBI), in whom the inflammation is thought to reflect immune-mediated mechanisms rather than clinically manifest TB disease [13]. TBD is defined as infection with microbiological detection of MTB and/or radiographic as well as clinical signs consistent with active disease, whereas TBI refers to MTB infection under persistent immune control without evidence of manifest disease [14].

The investigative approach to ocular TB is heterogeneous worldwide and often involves first identifying supportive ocular signs, followed by a step-wise approach of further “corroborating” investigations such as chest-imaging, immunologic investigations, and microbiological investigations including polymerase chain reaction (PCR) or mycobacteria growth indicator tube (MGIT) [15]. In this context, the QuantiFERON-TB Gold Test (QFT), an Interferon-Gamma-(IFN-γ)-Release-Assay, is firmly established as a pivotal diagnostic tool for detecting prior exposure to MTB. QFT is a whole-blood assay that measures the release of IFN-γ by T cells in response to MTB-specific antigens. The sensitivity of QFT varies from 62 to 95% (pooled value: 80%), and a specificity between 92 and 100% (pooled value: 98%) [16]. QFT detects prior exposure to MTB and does not differentiate between TBD and TBI.

Previously, routine TB screening in uveitis patients was recommended only in the presence of specific risk factors such as prior TB exposure, residence in endemic areas, or characteristic ocular findings like Eales disease, choroidal tuberculoma, and serpiginous-like choroiditis. The increased use of immunosuppressive therapies however, particularly tumour necrosis factor alpha (TNF-α) inhibitors, has necessitated routine TB screening to identify not only patients with TBD but also patients with TBI who are at risk of TB disease reactivation when exposed to immunosuppressive agents that block immune pathways that are pivotal for TB control [17, 18].

TB is known to cause uveitis in two different ways: First, there is the possibility of direct infection of the eye by MTB, typically through hematogenous spread of MTB rather than direct inoculation of the eye [19]. Here, focal granulomatous inflammation in uveal tissues reflects local host response to mycobacterial antigens, whether organism is viable or not [20]. Second, many patients have ocular inflammation without demonstrable organisms in the eye or elsewhere; this suggests delayed-type hypersensitivity or immune-mediated mechanisms to mycobacterial antigens that can trigger uveitis [21] which, as existing literature suggests, can develop in patients with TBD as well as TBI [13, 22].

Moreover, it is important to be aware that in clinical practice, the distinction between TBI and TBD relies on the negative predictive value of the investigations that are employed in order to rule out active disease. For example, a uveitis program that routinely uses more sensitive investigations like chest computed tomography (CT) scan and bronchoscopy to exclude TBD in QFT-positive patients with uveitis should likely detect more TBD cases than a program relying on X-ray, sputum, clinical symptoms, or uveitis phenotype alone. Current Collaborative Ocular Tuberculosis Study (COTS) consensus guidelines focus on a presumptive diagnosis of TBD based on ocular phenotype, immunologic testing, and radiographic evidence. In the COTS guidelines, the diagnostic utility of chest X-ray versus CT scan is not differentiated, and the recommendations do not include sputum culture, PCR, or bronchoscopy [23, 24].

This study aims to investigate the clinical utility of QFT in identifying patients with TBI as well as detecting TB-associated uveitis among uveitis patients in a tertiary care centre within a low endemicity context. Additionally, it seeks to highlight the nuanced presentation of tubercular uveitis, associated systemic findings, and diagnostic workup outcomes.

## Methods

### Study design and setting

This retrospective study analysed data from patients who presented to the Department of Ophthalmology with suspected uveitis and underwent QFT testing between 2013 and 2022. Diagnostic workup of uveitis patients at our tertiary uveitis clinic follows a standardized protocol, including laboratory testing with a complete blood count with differential, C-reactive protein (CRP) and/or erythrocyte sedimentation rate (ESR), creatinine, aspartate aminotransferase (AST), alanine aminotransferase (ALT), electrolytes, and syphilis serology. QFT testing is routinely performed in all uveitis patients, except in cases of isolated fibrinous anterior uveitis, where TB screening is conducted only if clinically indicated.

In uveitis cases with a positive QFT test as well as in cases of suspected sarcoidosis, serum soluble interleukin-2 receptor (sIL-2R) and angiotensin-converting enzyme (ACE) levels are measured, and a chest X-ray is performed. In cases with a suggestive ophthalmologic phenotype, an anterior chamber puncture is performed. Aqueous humour and paired serum samples are tested for pathogen-specific antibodies (including rubella virus, cytomegalovirus, herpes simplex virus, varicella zoster virus, and toxoplasma gondii). Intraocular antibody synthesis is evaluated using the Goldmann–Witmer coefficient. In cases of fibrinous inflammation, HLA-B27 typing is carried out. In patients with joint or gastrointestinal symptoms, additional rheumatologic or gastroenterological assessment is performed. Borrelia serology is conducted in patients with erythema migrans, arthritis, or clinical evidence of late neurologic manifestations. In intermediate uveitis occurring after the age of 45, a masquerade syndrome is excluded by cranial MRI, IL-10/IL-6 ratio analysis in anterior chamber fluid, or, if indicated, diagnostic vitrectomy. A cranial MRI is also obtained to exclude multiple sclerosis in patients with neurological symptoms. In cases of neuroretinitis, Bartonella serology is performed. For scleritis or retinal vasculitis with suspected systemic vasculitis or connective tissue disease, autoantibodies (ANA, ANCA, dsDNA) are determined. To rule out endophthalmitis in patients with chorioretinal infiltrates and corresponding clinical findings, blood cultures are taken and invasive diagnostics such as aqueous humour sampling, pars plana vitrectomy, or, if necessary, chorioretinal biopsy are performed. In cases of retinal vasculitis or focal retinitis with a history of oral aphthae and thus suspicion of Behçet’s disease, HLA-B51 typing is performed. HLA-A29 testing is carried out in cases with suspicion of birdshot uveitis [25].

Of note, QFT testing is part of the standardized diagnostic algorithm for patients with uveitis regardless of epidemiological or phenotypic risk factors for active TB. The QFT was employed either to identify the underlying cause of the inflammatory eye disease or to screen patients prior to initiating immunosuppressive therapy. Patients with a history of TB and/or previous treatment for TBI were excluded. Additionally, patients in whom uveitis could not be confirmed were also excluded from the study cohort. Data were collected from electronic medical records. The study was conducted in accordance with the Declaration of Helsinki and received approval from the local ethics committee (EA4/047/24).

### Study participants

The study included all patients presenting to the Department of Ophthalmology with confirmed uveitis and a positive QuantiFERON-TB test during the study period. Patients with a prior history of TB – regardless of treatment status – or those with a positive QuantiFERON-TB test but an ophthalmic diagnosis other than uveitis were excluded.

### QFT procedure

The QuantiFERON®-Tb-Gold Plus (QIAGEN, Hilden, Germany) is an enzyme-linked immunosorbent assay (ELISA) used to measure IFN-γ production by leukocytes after stimulation with MTB-specific peptides. For this purpose, heparinized blood samples were incubated for 24 h with the two MTB-specific proteins, ESAT-6 and CFP-10. The test determines whether the immune system has been previously exposed to MTB. A T1 or T2 value ≥ 0.35 IU/ml is considered a positive result, with a maximum reportable value of 10 IU/ml. QFT testing was performed prior to initiation or escalation of immunosuppressive therapy in the majority of cases. Patients already receiving high-dose corticosteroids (prednisolone equivalent ≥ 20 mg/day for ≥ 14 days) or disease-modifying anti-rheumatic drugs (DMARDs) at the time of QFT testing were noted, as immunosuppression may reduce QFT sensitivity. For these patients, negative QFT results were interpreted in conjunction with clinical, radiological, and ophthalmological findings.

### Data collection

The data collection for this study focused on both infectiological and ophthalmological aspects, ensuring a comprehensive assessment of patients undergoing QFT testing for uveitis.

#### Infectiological data

- **Clinical Symptoms**: Patients were assessed for B symptoms, including fever, night sweats, unintentional weight loss (> 10% over six months), and chronic cough (> 2 weeks).
- **HIV Status**: Documented to identify potential co-infection risks.
- **Pulmonary TB Diagnostics**:
  o Imaging: Chest X-rays and CT scans were reviewed for abnormalities such as lymphadenopathy, parenchymal changes, or pleural thickening/fluid accumulation.
  o Microbiological tests: Sputum samples (collected over three consecutive mornings) and bronchoalveolar lavage were analysed for MTB using cultural and microbiological methods.
- **TB Treatment**: Documentation included whether patients received standard anti-tubercular therapy for TBD or preventive therapy for TBI.
- **Immunosuppression**: Relevant immunosuppression was defined as:
  o Prednisolone equivalent ≥ 20 mg/day for ≥ 14 days.
  o Use of DMARDs, including biological agents

#### Ophthalmological data:

**Fig. 1:**
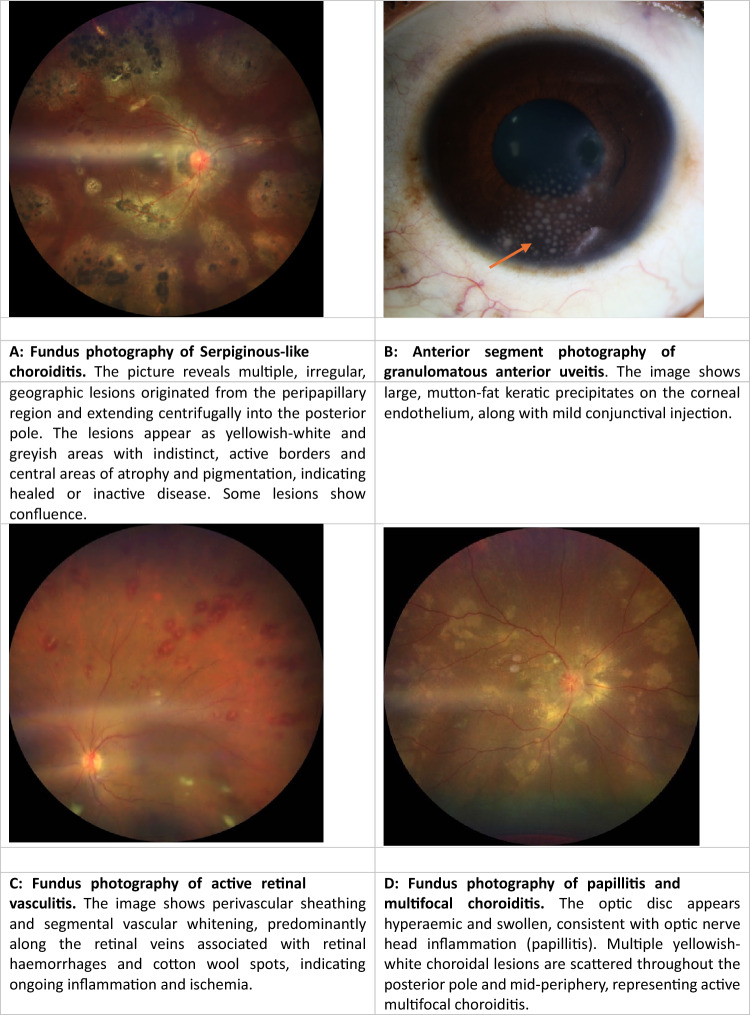
**A** Fundus photography of Serpiginous-like choroiditis. The picture reveals multiple, irregular, geographic lesions originated from the peripapillary region and extending centrifugally into the posterior pole. The lesions appear as yellowish-white and greyish areas with indistinct, active borders and central areas of atrophy and pigmentation, indicating healed or inactive disease. Some lesions show confluence. **B** Anterior segment photography of granulomatous anterior uveitis. The image shows large, mutton-fat keratic precipitates on the corneal endothelium, along with mild conjunctival injection. **C** Fundus photography of active retinal vasculitis. The image shows perivascular sheathing and segmental vascular whitening, predominantly along the retinal veins associated with retinal haemorrhages and cotton wool spots, indicating ongoing inflammation and ischemia. **D** Fundus photography of papillitis and multifocal choroiditis. The optic disc appears hyperaemic and swollen, consistent with optic nerve head inflammation (papillitis). Multiple yellowish-white choroidal lesions are scattered throughout the posterior pole and mid-periphery, representing active multifocal choroiditis

- Clinical Examination:
  o Visual acuity (VA) testing and slit-lamp examinations were conducted to assess the degree of ocular involvement.
- Classification of Uveitis:
- The SUN (Standardization of Uveitis Nomenclature) criteria were applied to define the anatomical location (anterior, intermediate, posterior, or panuveitis) and severity of inflammation (Fig. 1 A-D)
- Etiological Clarification:
- Additional investigations (e.g., anterior chamber puncture, blood analysis, rheumatology consultation) were performed to rule out other infectious or systemic causes as described in the Study setting section.

#### Diagnostic criteria

- The diagnosis of TBD in uveitis patients was based on the following criteria:
  i. positive culture result for MTB in any sample in or
  ii. any other microbiological evidence (PCR, stain) of MTB in any sample and/or
  iii. clinical presentation indicative of active tubercular uveitis according to the COTS criteria and decision to treat with a full course of ATT for 2 months RHZE (rifampicin, isoniazid, pyrazinamide, and ethambutol) followed by 4 months of RH (rifampicin and isoniazid).

#### Data sources

Data were extracted from electronic medical records.

This structured approach ensured a robust dataset, integrating clinical, diagnostic, and imaging information for a comprehensive evaluation of TB-associated uveitis.

### Statistical analysis

This retrospective cohort study used patient data extracted from medical records and entered into an Excel (Microsoft Excel, version 2019) database. All statistical analyses were performed with Stata 14 (StataCorp, College Station, TX). Continuous variables are presented as median and interquartile range (IQR) and categorical variables as number and percentage. Normality of continuous variables was assessed visually and by distributional checks; because many continuous variables were not normally distributed, comparisons of continuous variables between groups were made using the Mann–Whitney U test. Categorical variables were compared using the chi-square (χ^2^) test; when expected cell counts were small, Fisher’s exact test was applied. All hypothesis tests were two-sided and a *p*-value < 0.05 was considered statistically significant. Statistical estimates were reported with their corresponding 95% confidence intervals unless otherwise stated.

## Results

### Patient demographics and epidemiology

**Fig. 2:**
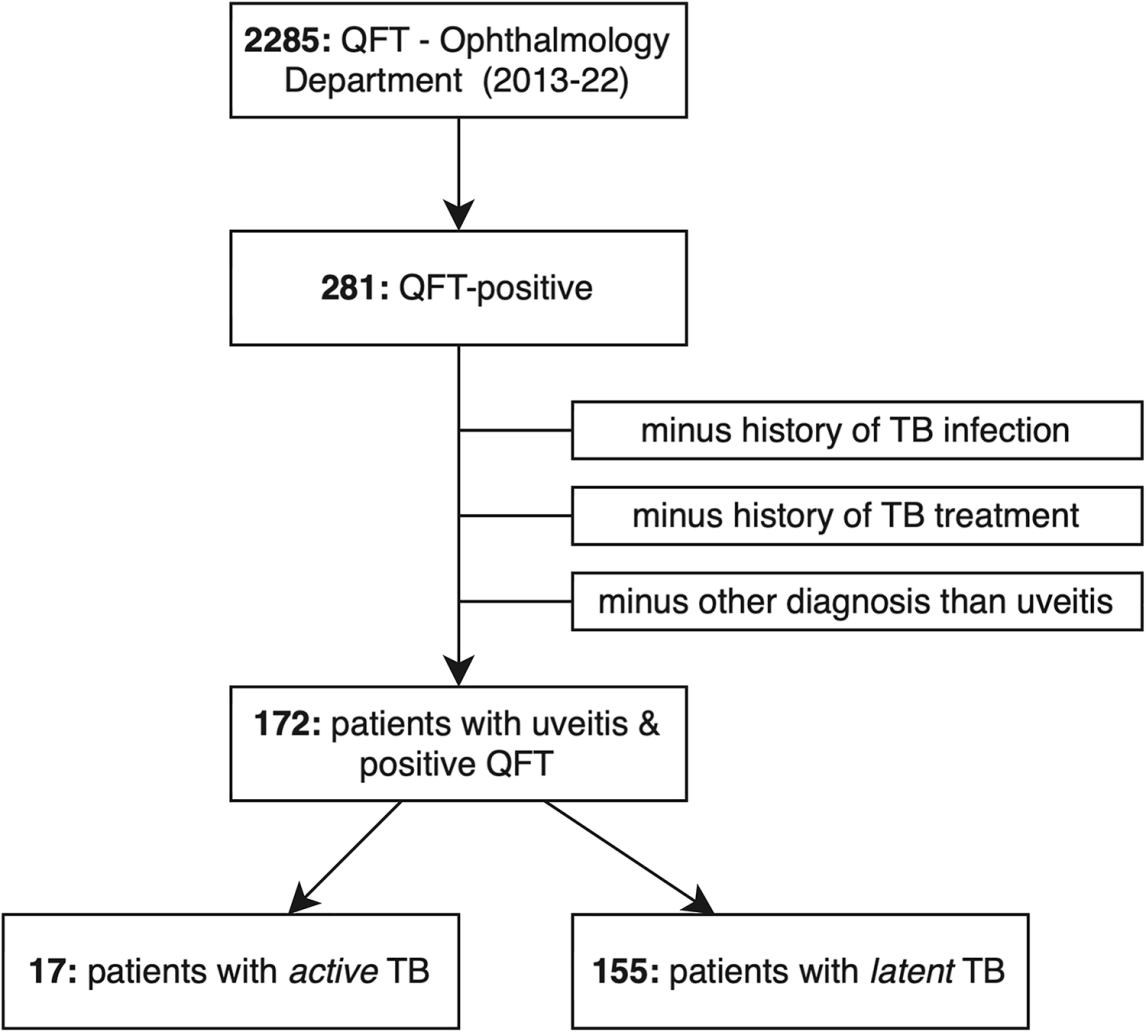
Flow chart illustrating the patient selection process and diagnostic pathway for the identification of uveitis patients with latent tuberculosis (TB) infection or active TB disease following QuantiFERON-TB Gold Test. Patients with a history or treatment of TB or unconfirmed uveitis were excluded. Final classification was based on clinical, radiological, and microbiological findings

A total of 2285 QFTs were analysed, of which 281 (12.3%) were positive. Of those 109 patients were excluded due to a history of prior TB disease or an ophthalmologic diagnosis other than uveitis. A total of 172 patients with uveitis and a positive QFT were included in the analysis. TBD was diagnosed in 9.9% (*n* = 17), while 90.1% (*n* = 155) were diagnosed with TBI (Fig. 2). Patients with TBD were significantly younger than those with TBI (median age: 40 vs. 58 years, *p* = 0.005). The majority of patients were male (56%), with no significant difference between TBD and TBI groups by sex (Table 1).

**Table 1:** Clinical and demographic characteristics of 172 patients with positive QFT and uveitis between Jan 2013 and Dec 2022

Variable		Total			TBD			TBI			*P*-value
		N	%(col)		N	%(row)		N	%(row)		
Total patients included		172	100		17	9.9		155	90.1		-
Age	Median (IQR)	56 (39–70)			40 (35–44)			58 (41–71)			**0.005**
Sex	Female	75		43.6	7		9.3	68		90.7	0.8
	Male	97		56.4	10		10.3	87		89.7	
Origin	GER	72	41.8		5		6.9	67		93.1	-
	AMR	2	1.2		0		0	2		100	-
	SEAR	16	9.3		9		56.3	7		43.7	** < 0.005**
	W-EURO	4	2.3		0		0	4		100	-
	EMR	9	5.2		1		11.1	8		88.9	0.7
	WPR	2	1.2		0		0	2		100	-
	E-EURO	29	16.9		0		0	29		100	0.1
	AFR	11	6.4		1		9.1	10		90.9	0.8
	Missing	27	15.7		1		3.7	26		96.3	-
HIV status	Negative	61	35.5		13		21.3	48		78.7	-
	Positive	3	1.7		1		33.3	2		66.7	0.6
	Not done	108	62.8		3		2.7	105		97.3	-
Clinical symptoms	Absent	165	95.9		14		8.5	151		91.5	-
	Present	5	2.9		3		60	2		40	** < 0.005**
	Missing	2	1.2		0		0	2		100	-
Number of uveitis episodes prior to first visit	No prior	119	69.1		11		9.2	108		90.8	-
	1	22	12.8		2		9.1	20		90.9	0.3
	2–10	29	16.9		3		10.3	26		89.7	
	> 10	2	1.2		1		50	1		50	
Duration [yrs] of illness prior to first visit (n = 53)	Median (IQR)	3.4 (1.8–7.6)			2.6 (0.5–4)			4.1 (1.8–7.6)			0.5
Immunosuppression prior to first visit	No	11	6.4		3		27.3	8		72.7	-
	Yes	27	15.7		4		14.8	23		85.2	0.4
	Unknown	134	77.9		10		7.5	124		92.5	-

GER, Germany, AMR, Americas, SEAR, South-East Asia Region, W-EURO, Western Europe, WPR, Western Pacific Region, E-EURO, Eastern Europe, AFR, African Region

*P*-values refer to comparisons between patients with TBD and TBI, using the chi-squared test for categorical variables and the Wilcoxon rank-sum test for continuous variables. *P*-values for missing data were not reported. For the variable origin, *p*-values refer to the odds of having a diagnosis of tuberculosis compared with the odds for patients of German origin. Clinical symptoms were defined as at least one of the following: cough > 2 weeks, unintended weight loss, night sweats or fever > 2 weeks. Duration of illness: only patients with a record of prior uveitis episodes were considered for this observation (*n* = 53). Two patients classified as tuberculosis infection were recorded as having clinical symptoms at initial presentation; after full workup, these symptoms were attributed to alternative causes by the attending physician at the time

Most QFT-positive patients originated from Germany and Eastern Europe, with 42% and 17% respectively. Notably, more than half of the patients diagnosed with TBD (56%) came from Southeast Asia. HIV testing was performed in 37% (*n* = 64) of patients. In total, 2% (*n* = 3) of patients were tested HIV positive. The prevalence of suggestive clinical symptoms was significantly higher in the TBD group (17.6% vs. 1.3%, *p* < 0.005). However, the majority of patients (*n* = 165; 96%) did not exhibit any systemic symptoms suggestive of TB. Most patients (69%) had no record of prior uveitis episodes. Of those with a record of prior uveitis episodes (*n* = 53), the median duration of disease was 3.4 years (IQR 1.9–7.6) with no significant difference between TBD and TBI groups (*p* = 0.5). Of note, 16% (*n* = 27) of patients had received immunosuppressive therapy for uveitis before presentation to our centre.

### Aetiologies of uveitis in patients with TBI

**Fig. 3:**
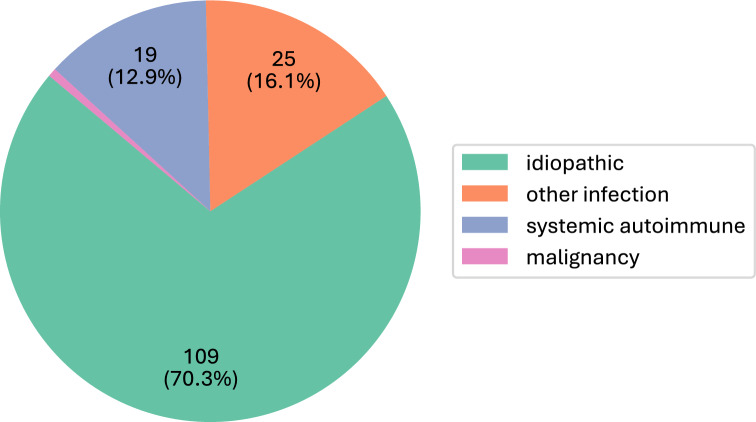
Aetiologies of uveitis in TBI patients (*n* = 155). Diagnosis categories: systemic autoimmune: confirmed diagnosis of autoimmune illness (e.g. sarcoidosis, rheumatoid arthritis, multiple sclerosis), other infectious uveitis: confirmed diagnosis of systemic infectious disease other than TB (e.g. toxoplasmosis, syphilis), malignancy: confirmed diagnosis of malignant disease (e.g. Lymphoma), idiopathic: uveitis without aetiological attribution to underlying disease

**Fig. 4:**
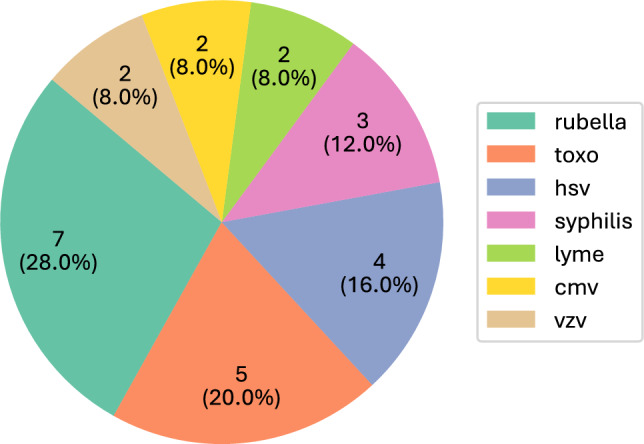
Infectious diseases aetiologies in TBI patients (*n* = 25): cmv, infection with cytomegalovirus; hsv, infection with herpes simplex virus; syphilis, infection with treponema pallidum; lyme, infection with borrelia species; rubella, infection with rubella virus; toxo, infection with toxoplasma gondii; vzv, infection with varicella-zoster virus

Among patients with a diagnosis of TBI (*n* = 155), idiopathic uveitis without any attributable cause was the most common uveitis classification (70.3%), followed by infectious aetiologies (16.1%) and autoimmune diseases (12.9%) (Fig. 3). We explored further the subset of patients with an infectious disease diagnosis other than TB (*n* = 25). Viral causes were most common (*n* = 15, 60%), followed by protozoal and bacterial causes (both *n* = 5, 20%). Among single infectious agents, rubella virus-associated (*n* = 7, 28%) and toxoplasmosis (*n* = 5, 20%) were most frequently detected (Fig. 4).

### Ophthalmologic characteristics

In total, 260 eyes of 172 patients were included, comprising 12.3% (32/260) with TBD and 87.7% (228/260) with TBI. Unilateral uveitis was present in 48.8% (*n* = 84) of patients and bilateral uveitis in 51.2% (*n* = 88) of patients. Among patients with TBD, bilateral presentation was significantly more common (*n* = 15, 88.2%) than among patients with TBI (*n* = 73, 47.1%; *p* < 0.005).

Among the anatomic phenotypes of uveitis, posterior uveitis was the most frequent (43.9%), followed by panuveitis (20%), intermediate (19.6%) and anterior uveitis (16.5%). Patients with TBD presented significantly more often with posterior uveitis than patients with TBI (*n* = 21 (65.5%) vs. *n* = 93 (40.8%); *p* = 0.03).

We characterized further the different ophthalmologic phenotypes. Retinal vasculitis was observed in 54 eyes (20.7%). Among patients with TBD, retinal vasculitis was significantly more common than among those with TBI (*n* = 23 (71.9%) vs. *n* = 31 (13.6%); *p* < 0.005). Serpiginous-like choroiditis was present in only 6% (*n* = 16), with no significant difference between TBD and TBI groups (*p* = 0.98). Multifocal chorioretinitis was observed in 30 eyes (3%) and was significantly more common in patients with TBD (*n* = 16 (50%) vs. *n* = 14 (6.1%; *p* < 0.005). Only one patient with TBD in the cohort was recorded as having iris nodules on examination (*p* = 0.007). Choroidal nodules were present in 61 eyes (23.5%) but was not associated with TBD (*p* = 0.82).

Visual impairment was slightly more severe in the TBD (0.5 logMAR: IQR 0.2–1) than the TBI group (0.4: IQR 0.2–0.7) without reaching statistical significance (*p* = 0.3). Table 2 summarizes the key details.

**Table 2:** Ophthalmologic characteristics of patients with uveitis and positive QuantiFERON-TB Gold Test

Variable		Total		TBD		TBI		*P*-value
		N	%(col)	N	%(col)	N	%(col)	
Total patients included		172	100	17	9.9	155	90.1	–
Total eyes included		260	100	32	12.3	228	87.9	–
Affected eyes (*n* = 172)	Unilateral	84	48.8	2	11.8	82	52.9	** < 0.005**
	Bilateral	88	51.2	15	88.2	73	47.1	
Uveitis	Anterior	43	16.5	2	6.3	41	18	–
	Intermediate	51	19.6	2	6.3	49	21.5	0.9
	Posterior	114	43.9	21	65.5	93	40.8	**0.03**
	Panuveitis	52	20	7	21.9	45	19.7	0.15
Retinal vasculitis	No	206	79.2	9	28.1	197	86.5	** < 0.005**
	Yes	54	20.7	23	71.9	31	13.5	
Serpiginous-like choroiditis	Yes	16	6	2	6.2	14	6.1	0.98
	No	244	94	30	93.8	214	93.9	
Multifocal chorioretinitis	Yes	30	11.5	16	50	14	6.1	** < 0.005**
	No	230	88.5	16	50	214	93.9	
Iris nodule	Yes	1	0.4	1	3.1	0	0	**0.007**
	No	259	9.6	3	96.9	228	100	
Choroidal nodule	Yes	61	23.5	7	21.9	54	23.7	0.82
	No	199	76.5	25	78.1	174	76.3	
Visual acuity [logMAR]	Median (IQR)	0.4 (0.2–0.7)		0.5 (0.2–1)		0.4 (0.2–0.7)		0.3

LogMAR, Logarithm of Minimum Angle of Resolution.

*P*-values were calculated for comparisons between patients with TBD and TBI using the chi-squared test for categorical variables and the Wilcoxon rank-sum test for continuous variables. P-values for missing data were not reported. For analysis of *affected eyes* patients (*n* = 172) were used instead of eyes (*n* = 260)

### Diagnostic features

In total, 105 sputum samples were performed on 48 (27.9%) patients. Among patients with TBD 15 of 17 (88%) had at least one sputum sample analysed during the study period. There were no sputum samples positive for MTB during the whole study period. A total of 13 patients (7.3%) underwent diagnostic bronchoscopy with or without endobronchial ultrasound (EBUS) guided lymph node biopsy. The bronchoscopy positivity rate was 31% (4/13, 3 EBUS and 1 BAL). Thus, only 24% (*n* = 4) of all TBD diagnoses were microbiologically confirmed (Table 3).

**Table 3:** Infectious disease diagnostic features in patients with TBI and TBD

Variable		Total		TBD		TBI		*p*-value
Total patients included		N	%(col)	N	%(row)	N	(%row)	-
		172	100	17	9.9	155	90.1	
Sputum	Positive	0	0	0	0	–	–	–
	Negative	48	27.9	15	31.25	33	68.75	–
	Not done	124	72.1	2	1.6	122	98.4	–
Bronchoscopy	EBUS +	3	1.7	3	100	–	–	–
	Lavage +	1	0.6	1	100	–	–	–
	Negative	9	5.2	2	22.2	7	77.8	–
	Not done	159	92.4	148	93	11	6.9	–
X-ray chest	Positive	22	12.8	6	27.3	16	72.7	0.08
	Negative	89	51.7	11	12.4	78	87.6	–
	Not done	61	35.5	0	0	61	100	–
CT scan chest	Positive	11	6.4	5	45.4	6	54.6	0.07
	Negative	15	8.7	2	13	13	86.7	–
	Not done	146	84.9	10	6.9	136	93.1	–

Sputum: non-induced morning sputum or sputum on spot; confirmed TB: cases with a microbiological result positive for MTB (either Ziehl–Neelsen stain, PCR or culture); clinical TB: cases without microbiological confirmation; EBUS: endobronchial ultrasound guided lymph node biopsy. Positive imaging findings include cavitation, (nodular) infiltrates, tree-in-bud pattern, hilar or mediastinal lymphadenopathy, pleuritis/pleural effusion

**Fig. 5:**
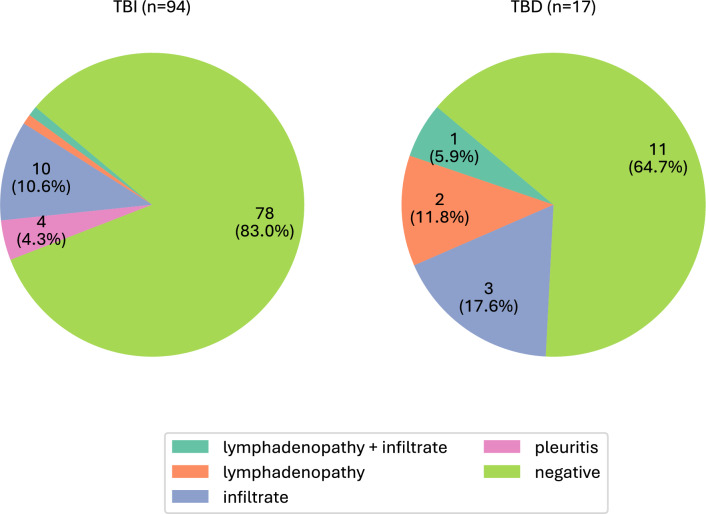
X-ray findings in uveitis patients with TB infection (TBI) vs. TB disease (TBD)

**Fig. 6:**
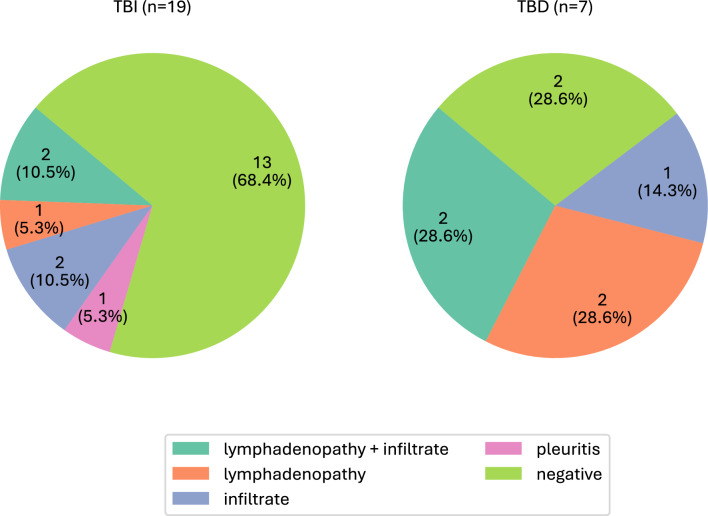
CT findings in uveitis patients with TB infection (TBI) vs. TB disease (TBD)

Chest X-ray was performed in 111 patients (65%) with 22 (20%) showing signs compatible with TB. While compatible chest X-ray findings were more common among the TBD than the TBI group, the trend did not reach statistical significance (*n* = 6 (35.3%) vs. *n* = 16 (17%); *p* = 0.08). Among the different x ray features (Fig. 5), lobar infiltrate was the most common among TBD patients (*n* = 3, 17.5%). CT of the chest was performed in 26 patients (15.1%) with 11 (42%) showing signs consistent with TB. Suspicious chest CT findings were observed more frequently in the TBD than in the TBI group; however, this difference did not reach statistical significance (*n* = 5 (71.4%) vs. *n* = 6 (31.6%); *p* = 0.07). Among the different suspicious features (Fig. 6), lymphadenopathy with or without infiltrate was most frequently detected among CT scans of TBD patients (*n* = 4 (57.2%). Chest X-ray and CT imaging demonstrated limited agreement. As an example, mediastinal lymphadenopathy was visible on CT in four TBD cases but was detected by chest X-ray in only one of them.

## Discussion

### Epidemiology

The prevalence of QFT positivity among our cohort of 2285 patients was 12.3% (281/2285). Among them, 172 patients presented with uveitis and had no prior diagnosis of TB (12.7%). Since QFT testing was not based on clinical suspicion but performed as part of a standardized diagnostic algorithm, this percentage is a robust estimate of the prevalence of QFT positivity among patients who present to a tertiary care centre in a large European city with suspected uveitis. Similar QFT positivity rates among uveitis patients have been reported in other non-endemic settings, including 16.6% in a French study [26] and 13% in a Dutch cohort [17].It is important to interpret these results in light of the diverse international background of patients presenting at tertiary centres such as ours. In our cohort, only 44% of patients originated from low-TB-prevalence countries, while 56% of those diagnosed with TBD originated from the WHO South-East-Asia (SEA) region. Notably, 53% (9/17) of all TBD cases originated from Southeast Asia, highlighting the importance of considering geographic background when assessing TB risk. While patients with clinical symptoms compatible with TB were significantly more likely to be diagnosed with TBD (60% vs 40%, p < 0.005), the overall prevalence of symptoms among TBD cases was very low (3/17; 18%). While these demographic factors contribute to the observed QFT positivity rates, they alone do not fully explain the association between TB exposure and ocular inflammation.

Increasing evidence suggests that even TBI with MTB can induce sustained immune activation, potentially influencing ocular immune homeostasis. Notably, elevated levels of certain autoantibodies in TBI patients suggest a link between infection and autoimmunity [27, 28]. Recent findings suggest that MTB may trigger autoimmune responses through molecular mimicry [29]. Correspondingly, elevated levels of autoantibodies have been observed in TB patients without underlying autoimmune disease [30]. These findings strongly support a potential immunopathogenic mechanism for ocular inflammation in patients without clinically active TB. Due to the low negative predictive values of current diagnostic methods, it remains difficult to distinguish autoimmune uveitis triggered by TBI from true ocular TBD presenting as uveitis.

### Diagnostic patterns in TBI versus TBD

#### Microbiology

In our cohort, TBD was diagnosed in 9.9% (17/172) of QFT-positive uveitis patients. Of these, only 23.5% (*n* = 4) were microbiologically confirmed. All microbiological confirmations (*n* = 4) were achieved using bronchoscopy with all, but one positive specimen recovered by EBUS guided fine needle aspiration (FNA) of mediastinal lymph nodes. None of the 105 sputum samples collected during the study period yielded positive results.

The microbiological diagnosis of TB uveitis remains challenging, as both culture and PCR testing frequently produce negative results in ocular fluids [30]. Although sputum collection, whether provoked or unprovoked, is minimally invasive, its sensitivity is low, and positivity is largely restricted to open pulmonary TB. Our findings support current COTS guidelines, which do not recommend sputum sampling for the diagnosis of tubercular uveitis.

Nevertheless, microbiological confirmation of TB remains important to reliably exclude alternative diagnoses and multidrug-resistant tuberculosis (MDR-TB). In high-prevalence regions of Europe, MDR-TB may account for up to 25% of newly diagnosed TB cases [31]. In such cases, more invasive procedures, such as bronchoscopy or EBUS guided FNA, provide greater diagnostic accuracy. In line with our results, a retrospective cohort study in a low prevalence setting in 2022 found a diagnostic yield of 90% was achieved with BAL and EBUS guided FNA among 49 TB patients with smear and TB-PCR negative sputum [32].

In clinical practice, the diagnosis of tubercular uveitis remains challenging, and most cases are established without microbiological confirmation. Instead, diagnosis generally relies on a composite approach that includes a compatible ocular phenotype, immunological evidence of TBI, exclusion of alternative aetiologies, and response to therapy [23, 24]. Although the invasiveness and associated risks of bronchoscopy must be balanced against its higher diagnostic yield, it can be a valuable diagnostic tool in selected patients with suspected TB uveitis who have imaging findings suggestive of active disease.

#### Imaging

A total of 65% (*n* = 111) of patients underwent X-ray, while 15.1% (*n* = 26) received chest-CT. Compared with TBI patients, those with TBD more frequently demonstrated pathological findings on both modalities—35.2% vs. 17% on chest X-ray and 71.4% vs. 31.7% on CT. Although this trend was more pronounced on CT, the differences did not reach statistical significance for neither X-ray (*p* = 0.08) nor CT (*p* = 0.07).

These findings are consistent with research in both the field of TB associated uveitis as well as TB diagnostic studies in general. Groen-Hakan et al. reported abnormal chest X-rays in 18.5% of a Dutch uveitis cohort [17], while Danjou et al. found pulmonary abnormalities in16% of QFT-positive patients [26]. More generally, in a large retrospective cohort study by Lau et al., chest CT was superior to chest X-ray in picking up pathologic changes in a cohort of patients with subclinical pulmonary TB [33], defined as a condition caused by live MTB that does not produce symptoms of active TB but results in detectable abnormalities on current radiologic or microbiologic tests. [34]. However, the lack of statistical significance in our comparison limits firm conclusions regarding imaging differences between TBD and TBI groups. It should also be acknowledged that CT was performed in a minority of patients and likely in those with higher clinical suspicion, introducing a potential selection bias. Imaging findings must therefore be interpreted within the broader clinical context, including symptoms, microbiological data, and immunological evidence of TBI. Furthermore, the detection of non-specific or subclinical imaging abnormalities carries the risk of overdiagnosis or overtreatment, and clinical relevance should guide the interpretation of any radiological finding.

#### *Ophthalmologic *f*eatures*

Among the recorded ophthalmologic phenotypes, three features were significantly more frequent among eyes of patients with TBD. Bilateral involvement occurred in 15 of 17 (88%) TBD patients compared with 72 of 155 (47%) TBI patients (*p* < 0.005). Posterior uveitis was the most common type (43.9%) and was significantly associated with TBD (*p* = 0.03). Retinal vasculitis occurred in 20.7% of eyes overall and was significantly more frequent in TBD than in TBI cases (65.6% vs. 40.7%; *p* < 0.005). Patients with TBD were slightly more affected than those with TBI, showing a trend toward greater visual impairment (0.5 vs. 0.4 logMAR; *p* = 0.3). The diagnostic value of these ophthalmologic features for distinguishing TBD from TBI should be interpreted with caution. The majority of TBD lacked microbiological confirmation. Hence, it is possible that features of disease severity such as bilateral eye involvement or severity of visual impairment themselves played a role in the clinical decision to initiate a full course of ATT. Therefore, using these features prospectively as a diagnostic criterion for the diagnosis of tubercular uveitis without microbiological confirmation would mean applying circular logic, thereby possibly overestimating the prevalence of these features in true cases of tubercular uveitis.

These findings highlight the diagnostic challenge of selecting appropriate tests while balancing yield, procedural risks, and radiation exposure. A practical approach is to establish pretest probability of TB: in low-probability cases, low-sensitivity tests may suffice, whereas in high-probability cases, negative results may be misleading. Currently, pretest probability is often assessed by ophthalmologic findings. Highly suggestive phenotypes, such as serpiginous-like choroiditis or tuberculomas, provide strong diagnostic clues, whereas nonspecific signs like retinal vasculitis are less discriminatory. Notably, the uveitis subtypes in this study did not clearly distinguish TBD from TBI.

In this context, the TB Flow assay, which analyses immunological markers in whole blood to differentiate TBD from TBI, shows strong potential to improve pretest probability estimates and guide the targeted use of invasive diagnostics like chest CT and bronchoscopy [35].

#### Aetiologies of uveitis

Among patients without a diagnosis of TBD, less than one-third (*n* = 46; 30%) had an identifiable alternative aetiology for their uveitis. No underlying disease was identified in 70% (*n* = 109) of patients with uveitis and TBI. These findings are consistent with a large European cohort study, where a higher proportion of uveitis cases with unknown aetiology was observed among QFT-positive compared to QFT-negative patients (59% vs. 39%) [17]. While this pattern may raise the possibility of undetected TBD in some cases, a positive QFT indicates immunological sensitisation to MTB but does not confirm active disease, particularly in a low-prevalence setting. In the absence of microbiological or radiological evidence, any inference regarding undiagnosed TBD must be made with caution.

### Limitations

This study had several limitations. As with all retrospective analyses of routine data, there were data quality issues that had the potential to skew single observations. For instance, the number of prior uveitis episodes was not routinely recorded for all patients so asking for prior uveitis episodes was at the discretion of the attending physician, potentially leading to underreporting. Additionally, some variables relevant to generalizability of our results were not consistently available. As mentioned above, the overall QFT positivity rate of 12.3% was calculated on the geographically heterogeneous population that presented to our clinic during the study period. A further analysis of the prevalence of QFT positivity by WHO region was not feasible, as detailed geographic data were not recorded in the database. Further, patients with negative QFT results did not undergo systematic TB-specific follow-up in this cohort. Data on TB disease occurring in IGRA-negative uveitis patients is therefore not available, which limits the completeness of TB ascertainment in this retrospective analysis. Moreover, intraocular PCR for MTB DNA detection was not part of the routine diagnostic algorithm during the study period, potentially leading to underdiagnosis of TB uveitis in some patients. Finally, the retrospective single-centre design inherently limits generalizability. Confirmation of these findings through prospective, multicentre studies with standardized diagnostic protocols and predefined outcome measures would substantially strengthen the evidence base.

## Conclusion

This study provides important real-world evidence on the prevalence and clinical relevance of positive QFT results in uveitis patients from a non-endemic setting. Despite low regional TB incidence, more than one in ten patients tested QFT-positive, emphasizing that latent or subclinical MTB remains a relevant consideration even in high-income countries. Our findings highlight the diagnostic challenge of distinguishing TBD from TBI in ocular inflammation. The limited sensitivity of conventional imaging and microbiological tests underscores the need for a structured, multidisciplinary approach that integrates immunologic, radiologic, and clinical data. Importantly, this study reinforces the role of QFT as a critical screening tool –both for uncovering TB-related uveitis and for preventing reactivation in patients receiving immunosuppressive therapy. Incorporating systematic TB screening into the uveitis workup, even in non-endemic regions, may reduce missed diagnoses and improve patient outcomes.

## Supplementary Information

Below is the link to the electronic supplementary material.

Supplementary file1 (DOCX 25 KB)

Supplementary file2 (DOCX 22 KB)

## Data Availability

Original data was extracted from patient records in the hospital database, then entered into a xcel database in an anonymised fashion. After database closure, the database was transferred into a stata format where statistical analysis was performed. On request, both the anonymised xcel database as well as the stata database can be provided.
