# Supplementary material for: QuantiFERON-TB gold test: a valuable diagnostic tool for tubercular uveitis in non-endemic regions
Source: Infection. 2026 Apr 17;54(3):1487–500. doi: 10.1007/s15010-026-02789-9 (PMC13323773; doi:10.1007/s15010-026-02789-9)
Supplement: Supplementary file 1 — Supplementary file1 (DOCX 25 KB) [file 15010_2026_2789_MOESM1_ESM.docx]

**Supplementary Table 1: Characteristics of 17 patients with clinically active tuberculosis disease**

| Pat ID | Origin | Age | Sex | Clinical symptoms | Disease duration [years] | Uveitis | X-ray chest findings | CT chest findings | Sputum | Bronchoscopy |
| --- | --- | --- | --- | --- | --- | --- | --- | --- | --- | --- |
| 1 | Sri Lanka | 29 | m | none | 1st episode | unilateral, posterior | none | none | 3*negative | not done |
| 2 | Pakistan | 52 | m | none | 14 | intermedia, bilateral | calcified parenchymal lesion in RUL | calcified mediastinal lymphadenopathy and calcified parenchymal lesion in RUL | 3*negative | TB PCR positive on EBUS |
| 3 | India | 28 | f | none | 4 | unilateral, panuveitis | none | none | not done | not done |
| 4 | India | 43 | m | none | 1st episode | bilateral, posterior | none | none | 3*negative | not done |
| 5 | Guinea | 19 | m | none | 1st episode | bilateral,  panuveitis | mediastinal and left hilar lymphadenopathy | non-calcified mediastinal and bihilar lymphadenopathy | 3*negative | EBUS and BAL negative |
| 6 | Sri Lanka | 40 | f | none | 1st episode | bilateral,  posterior | none | not done | 1*negative | not done |
| 7 | Vietnam | 56 | m | none | 1st episode | bilateral, posterior | none | not done | 1*negative | not done |
| 8 | Afghanistan | 35 | m | cough | 1st episode | bilateral, posterior | none | partially calcified mediastinal and bihilar lymphadenopathy | 3*negative | TB culture positive on EBUS |
| 9 | Germany | 43 | m | none | 1st episode | bilateral, posterior | none | not done | not done | not done |
| 10 | Germany | 43 | f | none | 2 | bilateral, anterior | none | not done | 1*negative | not done |
| 11 | Germany | 75 | m | none | 3 | bilateral, posterior | none | not done | 2*negative | not done |
| 12 | India | 31 | f | cough | 1st episode | bilateral, posterior | faint reticular infiltrate RUL and right hilar lymphadenopathy | not done | 3*negative | TB culture positive on EBUS, BAL negative |
| 13 | Germany | 72 | m | none | 1st episode | bilateral, posterior | right hilar lymphadenopathy | not done | 2*negative | not done |
| 14 | Indonesia | 37 | f | fever, cough | 1st episode | bilateral, panuveitis | bilateral miliary pattern | bilateral miliary pattern with postspecific complex in RUL | 3*negative | PCR positive on BAL |
| 15 | unknown | 37 | f | none | 1st episode | bilateral, panuveitis | none | none | 1*negative | not done |
| 16 | Vietnam | 38 | m | none | 0.5 | bilateral, posterior | faint opacity in RUL | postspecific complex in RUL and non-calcified mediastinal lymphadenopathy | 1*negative | BAL negative, EBUS with non-caseating granuloma; no TB PCR performed on EBUS sample |
| 17 | Germany | 44 | f | none | 4 | bilateral, posterior | none | not done | 3*negative | not done |

Clinical symptoms: generalized symptoms compatible with active tuberculosis; Disease duration: Length of uveitis associated symptoms before presentation to our ophthalmology clinic; RUL: Right upper lobe; CT: Computed Tomography; TB: Tuberculosis; PCR: Polymerase Chain Reaction; EBUS: Endobronchial Ultrasound; BAL: Bronchoalveolar Lavage.

**Supplementary Table 2: Ophthalmological features of 17 patients with clinically active tuberculosis disease**

| Eye ID | Uveitis | Visual acuity [logMAR] | Anterior keratic precipitates | Iris nodules | Vitreous haze | Snowballs | Snowbank | Multifocal chorio-retinitis | Serpiginous-like choroiditis | Choroidal nodule | Papillitis | Retinal Vasculitis | Macular edema |
| --- | --- | --- | --- | --- | --- | --- | --- | --- | --- | --- | --- | --- | --- |
| 1r | pos | 0.4 | - | - | - | - | - | yes | - | - | yes | yes | - |
| 2r | int | 0.5 | g | - | 1 | - | - | - | - | - | - | yes | yes |
| 2l | int | 0.4 | ng | - | 1 | - | - | - | - | - | - | yes | yes |
| 3l | pan | 1.9 | g | - | 3 | - | - | - | - | - | yes | yes | yes |
| 4r | pos | 0.1 | - | - | - | - | - | yes | - | yes | - | yes | - |
| 4l | pos | 1.9 | g | - | - | - | - | yes | - | yes | - | yes | - |
| 5r | pan | 0.4 | - | - | - | - | - | yes | - | yes | yes | yes | yes |
| 5l | pan | 0.3 | - | - | - | - | - | yes | - | yes | yes | yes | yes |
| 6r | pos | 0.6 | - | - | 0.5 | - | - | - | - | - | yes | yes | yes |
| 6l | pos | 0.2 | - | - | 1 | - | - | - | - | yes | - | yes | - |
| 7r | pos | 0.1 | - | - | - | yes | - | - | - | - | yes | yes | yes |
| 7l | pos | 0.2 | - | - | - | yes | - | - | - | - | yes | yes | - |
| 8r | pos | 1.2 | - | - | - | - | - | yes | - | - | yes | yes | yes |
| 8l | pos | 0.1 | - | - | - | - | - | yes | - | - |  | yes | yes |
| 9r | pos | 0.7 | - | - | - | - | - | yes | - | - | yes | yes | yes |
| 9l | pos | 0.5 | - | - | - | - | - | yes | - | - | - | yes | - |
| 10r | ant | 0.1 | - | - | - | - | - | - | - | - | - | - | - |
| 10l | ant | 0.1 | - | yes | - | - | - | - | - | - | - | - | - |
| 11r | pos | 0.6 | - | - | - | - | - | yes | yes | - | - | - | - |
| 11l | pos | 0.4 | - | - | - | - | - | yes | yes | - | - | - | yes |
| 12r | pos | 0.2 | - | - | - | - | - | - | - | - | yes | yes | yes |
| 12l | pos | 1.3 | - | - | - | - | - | yes | - | - | yes | yes | yes |
| 13r | pos | 0.6 | - | - | - | - | - | yes | - | - | yes | - | yes |
| 13l | pos | 0.3 | - | - | - | - | - | yes | - | - | yes | - | Yes |
| 14r | pan | 1.6 | ng | - | 3 | yes | yes | - | - | - | - | yes | - |
| 14l | pan | 1.9 | ng | - | 3 | - | yes | - | - | - | yes | yes | - |
| 15r | pan | 1 | g | - | 1 | - | - | yes | - | yes | yes | yes | yes |
| 15l | pan | 1 | g | - | 0.5 | - | - | yes | - | yes | yes | yes | yes |
| 16r | pos | 0.6 | g | - | 1 | - | - | - | - | - | yes | yes | yes |
| 16l | pos | 0 | - | - | - | - | - | - | - | - | - | Yes | - |
| 17r | pos | 1 | - | - | - | - | - | - | yes | - | - | - | yes |
| 17l | pos | 1 | - | - | - | - | - | - | yes | - | - | - | - |

Uveitis: ant: anterior, int: intermedia, pos: posterior; Anterior keratic precipitates: g: granulomatous precipitates, ng: non-granulomatous precipitates; Vitreous haze grading: 0: nil, 0.5: trace, 1: minimal, 2: mild, 3: moderate, 4: marked, 5: severe.
