# Supplementary material for: QuantiFERON-TB gold test: a valuable diagnostic tool for tubercular uveitis in non-endemic regions
Source: Infection. 2026 Apr 17;54(3):1487–500. doi: 10.1007/s15010-026-02789-9 (PMC13323773; doi:10.1007/s15010-026-02789-9)
Supplement: Supplementary file 2 — Supplementary file2 (DOCX 22 KB) [file 15010_2026_2789_MOESM2_ESM.docx]

**Title page**

Title:

**QuantiFERON-TB Gold Test: A Valuable Diagnostic Tool for Tubercular Uveitis in Non-Endemic Regions**

Author information

**Sebastian Albus ^1*^**

^1^ Charité – Universitätsmedizin Berlin, corporate member of Freie Universität Berlin and Humboldt- Universität zu Berlin, Fächerverbund für Infektiologie, Pneumologie und Intensivmedizin, Augustenburger Platz 1, 13353 Berlin, Germany

Email: [sebastian.albus@charite.de](mailto:sebastian.albus@charite.de)

ORCID-Nr.: 0000-0002-4655-598X

**Antonia Koch ^2^**

^2^ Charité *–* Universitätsmedizin Berlin, corporate member of Freie Universität Berlin and Humboldt- Universität zu Berlin, Department of Ophthalmology, Augustenburger Platz 1, 13353 Berlin, Germany

**Uwe Pleyer ^2,3^**

^2^ Charité *–* Universitätsmedizin Berlin, corporate member of Freie Universität Berlin and Humboldt- Universität zu Berlin, Department of Ophthalmology, Augustenburger Platz 1, 13353 Berlin, Germany

^3^ Berlin Institute of Health at Charité – Universitätsmedizin Berlin, Charitéplatz 1, 10117 Berlin, Germany

ORCID-Nr.: 0000-0002-5569-1473

**Anne Rübsam ^2^**

^2^ Charité *–* Universitätsmedizin Berlin, corporate member of Freie Universität Berlin and Humboldt- Universität zu Berlin, Department of Ophthalmology, Augustenburger Platz 1, 13353 Berlin, Germany

ORCID-Nr.: 0000-0001-6202-1710

**Leif E. Sander ^1^**

^1^ Charité – Universitätsmedizin Berlin, corporate member of Freie Universität Berlin and Humboldt- Universität zu Berlin, Fächerverbund für Infektiologie, Pneumologie und Intensivmedizin, Augustenburger Platz 1, 13353 Berlin, Germany

ORCID-Nr.: 0000-0002-0476-9947

**Martin Witzenrath ^1^**

^1^ Charité – Universitätsmedizin Berlin, corporate member of Freie Universität Berlin and Humboldt- Universität zu Berlin, Fächerverbund für Infektiologie, Pneumologie und Intensivmedizin, Augustenburger Platz 1, 13353 Berlin, Germany

ORCID-Nr.: 0000 0002 9787 5633

**Nikolai Menner ^1^**

^1^ Charité – Universitätsmedizin Berlin, corporate member of Freie Universität Berlin and Humboldt- Universität zu Berlin, Fächerverbund für Infektiologie, Pneumologie und Intensivmedizin, Augustenburger Platz 1, 13353 Berlin, Germany

**Lynn S. zur Bonsen ^2#^**

^2^ Charité *–* Universitätsmedizin Berlin, corporate member of Freie Universität Berlin and Humboldt- Universität zu Berlin, Department of Ophthalmology, Augustenburger Platz 1, 13353 Berlin, Germany

**Dominika Pohlmann ^2,3*#^**

^2^ Charité *–* Universitätsmedizin Berlin, corporate member of Freie Universität Berlin and Humboldt- Universität zu Berlin, Department of Ophthalmology, Augustenburger Platz 1, 13353 Berlin, Germany

^3^ Berlin Institute of Health at Charité – Universitätsmedizin Berlin, Charitéplatz 1, 10117 Berlin, Germany

* Corresponding Authors

^#^ These authors contributed equally to this work and should be considered joint last authors
